# Australian hospital staff perceptions of barriers and enablers of domestic and family violence screening and response

**DOI:** 10.1186/s12913-021-07083-y

**Published:** 2021-10-19

**Authors:** Debra K. Creedy, Kathleen Baird, Kerri Gillespie, Grace Brandjerdporn

**Affiliations:** 1 Transforming Maternity Care Collaborative, School of Nursing and Midwifery, Griffith University, University Drive, Meadowbrook, Queensland 4131 Australia; 2 Gold Coast University Hospital, Parklands Drive, Meadowbrook, 4215 Australia; 3 Centre for Midwifery, Child and Family Health, School of Nursing and Midwifery, Faculty of Health, University of Technology Sydney, Ultimo, 2007 Australia

**Keywords:** Domestic and family violence, Hospital staff, Survey, Screening, Clinical practice

## Abstract

**Background:**

Hospital presentations provide unique opportunities to detect DFV. However, up to 70% of women experiencing Domestic and Family Violence (DFV) go undetected by hospital staff. While routine DFV screening is internationally encouraged, there is still much debate surrounding its implementation. The aim of the study was to determine staff perceptions of barriers and enablers of DFV screening and response.

**Methods:**

A cross-sectional survey was conducted at a tertiary level public hospital and health service. Health care staff in allied health, maternity and mental health divisions (*n* = 615) were invited to participate by email and through team meetings. 172 responses were analysed.

**Results:**

Less than a third of respondents reported routinely asking patients about DFV, with 34.9% reporting they did not have sufficient training to assist with DFV. Increased levels of training were positively correlated with screening practices, preparedness and knowledge. Major barriers were presence of partner and language barriers, while written protocols and supportive work environment were the principal enablers of screening.

**Conclusion:**

Staff generally believed that routine screening was important and should encompass all forms of abuse. Many felt ill-equipped to ask about or manage disclosure of DFV. More training improves staff capacity for DFV detection and response, and written guidelines should be made available to all staff.

## Background

Domestic and Family Violence (DFV) encapsulates a wide array of abuse and acts of coercion or intimidation within a domestic setting, that violate liberty and aim to establish control over the victim [World Health Organisation (WHO), 2013]. DFV is usually perpetrated by a known male against a woman. The lifetime prevalence of DFV for women globally is around one in three [1]. In Australia, one in six women have experienced physical or sexual violence from a current or previous partner, with one woman killed every 9 days [2].

Almost a third of hospitalisations due to assault are attributed to DFV, yet it is estimated that up to 70% of women experiencing DFV go undetected by hospital staff [2]. Of women killed by a partner, around 40% were seen by a health care provider in the preceding 12 months [3]. Given these statistics, visits to health care agencies provide unique opportunities to detect risk or existing DFV and provide care, referral, and/or emergency planning for women.

Research consistently reports that both staff and women value routine DFV screening in clinical practice [4–6]. However, there is no consistency in screening across healthcare services, and those that have implemented routine inquiry often do so without adequate ongoing training, processes and available resources [7]. Studies show that training for staff can greatly increase the occurrence of screening as well as detection [8]. There is also evidence from studies within General Practice clinics, that screening and detection can lead to improvements in health and wellbeing of women ([9].

Health care services are increasingly encouraged to offer comprehensive training for staff and make adequate system changes for routine DFV screening and support [10, 11]. Despite the enormous personal, social, health and economic costs of DFV, and wealth of research dedicated to this issue, there remains insufficient evidence regarding the best way to address DFV within healthcare services. Research has yet to conclude best practice for DFV screening (detection) and response [12, 13]. The overall aim of the study was to determine staff perceptions of barriers and enablers of DFV screening and response using the following the research questions:

1. To what extent does clinicians’ preparedness and knowledge hinder or enable frequency of DFV screening and response?
2. What do clinicians perceive as the main barriers to DFV screening and response?
3. What do clinicians perceive as the main enablers to DFV screening and response?
4. What are the differences among professional groups in regard to DFV practices?

## Methods

### Design

A cross-sectional survey design was used.

### Participants and setting

Employees in the allied health, maternity and mental health divisions who provided direct patient care at a large tertiary hospital in Queensland were invited by email or during team meetings to participate in an anonymous online or paper-based survey. Participants were all registered health professionals, 18 years or older, with an ability to speak and write English.

### Survey

The survey was developed from staff questionnaires routinely used at the participating site and a review of current and existing literature. The survey consisted of: (1) a brief demographic section; (2) perceptions of preparedness to conduct DFV screening and response (Baird et al); (3) DFV knowledge scale (Baird et al); (4) frequency of DFV screening; (5) frequency of DFV response practices; and open ended questions asking about barriers and enablers of DFV screening. A description of scales with example items and Cronbach’s alpha coefficients are presented in Table 1. The survey was conducted between June 2019 and October 2019.

**Table 1:** Survey content, internal reliability, and approach to analysis

Scale and Cronbach’s alpha	Description and item examples	Approach to analysis
Preparedness α = 0.98	21 items about how prepared respondents were to perform specific tasks related to screening. Response scale of 1 = not prepared to 5 = well prepared. Questions included: *Ask appropriate questions about DFV; help a woman create a safety plan; document in a women’s chart;* and *know what questions to ask.*	Scores summed; descriptive statistics; Correlation with other scales (Spearman’s rho) ANOVA + post-hoc test (professional groups x level of preparedness)
Knowledge α = 0.72	Respondents were asked to answer *True, False,* or *don’t know* to 20 questions including: *There are common, non-injury presentations of DFV (true); There are no good reasons for not leaving a violent relationship (false);* and *strangulation injuries are rare in cases of DFV (false).*	Scores summed; descriptive statistics; Correlation with other scales (Spearman’s rho) Kruskal Wallis tests + post-hoc test (professional groups x knowledge level)
Screening Practices α = 0.72	13 questions about how often respondents screen for DFV on a scale of 1 = never, to 5 = always. Questions included: *I routinely ask a woman about her home life; I provide education to women about the short and long-term effects of trauma; I carry out a brief mental health assessment.*	Scores summed; descriptive statistics; Correlation with other scales (Spearman’s rho). Kruskal Wallis tests + post-hoc test (professional groups x screening practices. Two categories “never, seldom, sometimes’ and ‘almost always/always’
Response Practices α = 0.85	10 questions on how often respondents performed specific tasks when they identified DFV on a scale of 1 = never, to 5 = always. Questions included: *used a body map to document injuries; conducted a safety assessment;* and *referred a woman to community domestic violence services.*	Correlation with other scales (Spearman’s rho). Kruskal Wallis tests + post-hoc test (professional groups x response practices. Two categories “never, seldom, sometimes’ and ‘almost always/always’
Barriers and enablers to screening	17 items on 5 point scale from 1 = ‘very limiting for me’ to 5 = ‘very helpful for me’ including ‘*My case/work load is too high’ and ‘Having a written DFV screening protocol in the department’*.	Responses coded as a barrier or enabler. Frequency of issues reported.

### Data analysis

Survey results were analysed using SPSS version 26 (IBM, 2019). The approach to analysis is shown in Table 1. Descriptive statistics were used to analyse personal/professional details. Total scale scores were calculated. Scale reliability was determined using Cronbach’s alpha. Differences between professional groups were analysed using ANOVA, or Kruskal Wallis where homogeneity of variance was violated. Correlations were performed using non-parametric Spearman’s rho tests. Post hoc analyses were conducted using Tukey’s tests.

### Ethics

Participation in the study was voluntary and confidentiality was assured to all participants and no personal identifiable was included. Completion of the survey implied consent. Ethical approval was obtained from the Gold Coast University Hospital Human Research Ethics Committee (HREC/15/QGC/87).

## Results

Around 615 staff were invited to complete the survey. The survey used to collect the data was developed from staff questionnaires routinely used at the participating site during staff training. After data cleaning, 172 surveys were entered into the analysis giving a response rate of 27.9%. Respondents were predominantly female nurses, midwives, medical staff or social workers with a mean age of 44.6 years (see Table 2). The extent of clinical experience varied considerably with the average being 11.5 years. Just under half of the respondents (46%) had personally experienced DFV at some time.

**Table 2:** **Respondent characteristics**

Item	N (%)	Mean (SD)
**Role**		
Midwife	42 (24.4)	
Nurse	51 (29.7)	
Medical Officer	34 (19.8%)	
Social Worker	30 (17.4)	
Psychologist	5 (2.9)	
Other Allied Health	3 (1.7)	
Other	7 (4.1)	
**Years in clinical practice**	172	13.42 (11.14) Range = 0–45
**Age**	168	44.67 (11.56) Range = 21–66
**Gender**		
Male	23 (13.4)	
Female	144 (83.7)	
**Have you ever personally experienced DFV?**		
No	88 (53.3)	
Yes	77 (46.7)	
**Are you currently experiencing DFV?**		
No	126 (73.3)	
Yes	6 (3.5)	
**Previous hours of DFV-related training**	155	18.9 (35.72) Range = 0–300

### Knowledge

When asked a series of knowledge questions relating to DFV, no participant scored all 20 items correctly which included questions on DFV presentation, injury patterns, contributors and patient trauma. The mean score was 12.9 out of a possible 20 (SD 3.56, range = 0–19). Almost a quarter of respondents (22.1%, *n* = 38) scored 10 or less items correctly.

### Perceived level of preparedness

Respondents had a mean score of 63.6 out of a possible 105 (SD 22.6, range = 21–105) when questioned about perceived preparedness to screen, respond to DFV, conduct safety assessments and safety plans, or make appropriate referrals and documentation. Around 45 % of respondents stated they felt “fairly well or well prepared” to conduct most of these tasks. Thirty-five percent reported being “moderately, prepared” while around 20 % of participants reported being minimally or not prepared.

### Routine DFV screening practices

Regarding practices, participants generally scored better on detection (indicating they routinely questioned women on DFV related issues, established rapport and provided information), than on response practices (photographing injuries, conducting safety assessments, and making appropriate notification and referrals). However, fewer participants answered these questions (*n* = 110 for screening questions; *n* = 78 for responding questions). These low rates may reflect the number of clinicians in managerial roles, allied health and ‘other’ participants, who were not commonly expected to conduct screening or perform clinical procedures (such as a physical examination) should DFV be detected.

Of the 110 participants who responded, only 10.9% (*n* = 12) answered “sometimes, seldom, or never” to the screening practices outlined in the survey (see Table 3). Almost 30 % (29.1%, *n* = 32) reported that they ‘nearly always’, or ‘always’, used these screening techniques, with the rest scoring an average rating of between ‘sometimes’ and ‘nearly always’. The scale mean score was 47.86 (SD 6.67, range = 23–62). Around 30 % (30.2% *n* = 52) reported always asking all women about a history of DFV in their relationships. Whereas a similar proportion (29.7% *n* = 51) thought they could identify DFV without asking specific questions. Around 61% (*n* = 105) reported being able to make appropriate referrals to services within the community for women experiencing DFV.

**Table 3:** Screening practices - scale and item scores

Scale Mean 47.86 SD 6.67, range = 23–62		
Item	Never, Seldom, Sometimes N (%)^a^	Almost always, always N (%)
1. I routinely ask a woman about her home life	27 (24.5)	83 (75.5)
2. I do a head to toe check for signs of physical abuse	96 (87.3)	14 (12.7)
3. I tend not to ask about sexual abuse	94 (85.5)	16 (14.5)
4. I try to establish rapport with a woman, and then ask questions to determine if she is at risk in the home or elsewhere	20 (18.2)	90 (81.8)
5. I provide education to women about the short and long-term effects of trauma	55 (50.0)	55 (50.0)
6. I carry out a brief mental health assessment	23 (20.9)	87 (79.1)
7. I tend not to ask the partner and other accompanying adults to excuse themselves from the room	96 (87.3)	14 (12.7)
8. I make rapport with a woman by looking directly at her when asking them questions about DFV	58 (52.7)	52 (47.3)
9. I adapt my approach according to a woman’s experience of DFV and her history of trauma	15 (13.6)	95 (86.4)
10. If the woman presents with children, I try and separate the children from their mother when I ask her questions about DFV	36 (32.7)	74 (67.3)
11. I discuss cases of women at risk wit another member of staff	18 (16.4)	92 (83.6)
12. I call the domestic violence hotline for advice	83 (75.5)	27 (24.5)
13. I ask the social worker to assess the woman.	41 (37.3)	69 (62.7)

^a^ Percentages reflect those who responded, not percentage of total sample

### Self-reported response practices

Less than half of all respondents (*n* = 78) were able to answer these questions due to their clinical position, while others reported these questions were not relevant to their practice (see Table 4). The scale mean score was 33.62 (SD 8.52, range = 10–50). Thirty percent of participants (30.8%, *n* = 24 of those responding to these questions) reported an average rating of ‘sometimes, seldom, or never’ for these response practices. Twenty participants (25.6%) scored an average of ‘nearly always or always’ when asked about the frequency with which they conducted DFV response practices. Remaining participants scored an average of 3 to 4 (between sometimes and nearly always) on all 13 items.

**Table 4:** Response Practices - Scale and item scores

Scale Mean 33.62 SD 8.52, range = 10–50		
Item	Never, Seldom, Sometimes N (%)	Almost always, always N (%)
1. Documented the woman’s statements about DFV in her medical record	85 (49.4)	87 (50.6)
2. Used a body map to document injuries	164 (95.3)	8 (4.7)
3. Photographed a woman’s injuries to include in her medical record	170 (98.8)	2 (1.2)
4. Notified appropriate authorities when required	104 (60.5)	68 (39.5)
5. Conducted a safety assessment with the woman	102 (59.3)	70 (40.7)
6. Conducted a safety assessment for the woman’s children	101 (58.7)	71 (41.3)
7. Sought advice from another work colleague	98 (57.0)	74 (43.0)
8. Discussed the case with / referred the case to Social Work	98 (57.0)	74 (43.0)
9. Referred a woman to community domestic violence services	116 (67.4)	56 (32.6)
10. Contacted a DFV service provider on behalf of a woman	132 (76.7)	40 (23.3)

Percentages reflect those who responded, not percentage of total sample

### Factors enabling or hindering DFV screening and response

Analyses were conducted according to the dominant professional groups of respondents (midwives, nurses, medical officers and social workers). Levels of preparedness were significantly different between groups (F (3, 139) = 22.57, *p* < .001). Post hoc tests showed that social workers were significantly more prepared than all other groups (mean = 4.18; *p* < .001) and nurses were significantly less prepared than all other groups (mean = 2.44; *p* < .01). Kruskal Wallis tests showed a significant difference between groups for knowledge (χ^2^ (3,139) = 33.362, *p* < .001), with social workers scoring the highest (mean = 14.96), followed by midwives (mean = 14.36), medical officers (mean = 12.44), and nurses (mean = 11.29). Screening practices (χ^2^ (3,139) = 9.011, *p* = .029) also showed differences between professional groups with social workers scoring the highest frequency of practices (mean = 49.65), followed by midwives (mean = 49.53), medical officers (mean = 46.7) and nurses (mean = 43.9).

#### Training

Increases in hours of training positively correlated with higher scores on three scales (Perceived Preparedness, *r =* 0.56*, p* < .01; Knowledge, *r* = 0.38, *p* < .01 and Screening practices, *r* = 0.42, *p* < .01). A Kruskal-Wallis test indicated that levels of training differed significantly between the four major professional groups (χ^2^ (3, 139) = 31.87, *p* < .001), with social workers receiving the largest number of training hours (mean = 40.96, SD = 46.15) followed by midwives (mean = 18.15, SD = 19.34), nursing staff (mean = 14.02, SD = 44.91) and medical officers (mean = 13.14, SD = 23.17).

#### Barriers and enablers to screening

Perceived Preparedness and Knowledge were conceptualised as enablers of DFV screening and response. There was a high positive correlation between Perceived Preparedness and Knowledge scores (*r* = 0.39, *p* < .01), and Screening practices (*r* = 0.27, *p* < .01), but no relationship with DFV Response practices. Those who performed well for Screening practices also performed well for Response practices (*r* = 0.66, *p* < .01).

Most respondents (*n* = 159, 92.4%) reported that clinicians should screen for all types of abuse (including sexual, physical, and psychological abuse, threats, stalking, economic control and intimidation). Two (1.2%) respondents felt clinicians should not screen for any types of abuse.

Tables 5 and 6 outline those factors respondents felt were limiting or helpful when screening for DFV. Major barriers to screening were the presence of the woman’s partner, language barriers, lack of rapport with the woman, and lack of a comfortable/private room for DFV questions (see Table 5). Enablers to screening and response included having support from colleagues and the organisation, as well as the presence of written protocols and guidelines (see Table 6). Seventy percent (69.8%, *n* = 120) felt encouraged by their workplace to respond to DFV.

**Table 5:** Major barriers to screening (*N* = number of respondents who consider this a limiting factor)

Barriers	N (%)
No interpreter available	151 (87.8)
Presence of woman’s partner	145 (84.3)
Woman doesn’t speak English fluently	137 (79.7)
Not having built rapport with the woman	131 (76.2)
No comfortable space to speak with women about DFV	125 (72.7)
No social worker available	125 (72.7)
No single rooms available	105 (61.0)
My workload is too high	84 (48.8)

**Table 6:** Enablers to screening (*N* = number of respondents who consider this an enabling factor)

Enablers	N (%)
A belief in screening for all types of abuse	159 (92.4)
Working in an environment that advocates and prioritises self-care	131 (76.2)
Having a written DFV screening protocol	125 (72.7)
Having confidence to discuss DFV with women	128 (74.4)
My workplace encourages me to respond to DFV	120 (69.8)
My colleagues are supportive of DFV screening	119 (69.2)
Having an online response protocol on what to do if I suspect or detect DFV	117 (68.4)
Knowledge of other services/organisations that support women experiencing DFV	112 (65.1)

## Discussion

This survey of hospital staff illustrated a modest degree of perceived preparedness and knowledge by clinicians to screen and address DFV in the clinical setting. Overall, levels of preparedness were somewhat lower than expected given that regular DFV training had been offered at the participating site since 2015 [8]. Compared to other professional groups, social workers felt more prepared to ask questions about DFV. This is unsurprising given their professional training and role in assessing and supporting people with psychosocial risks or in crisis. Midwives also showed a comparatively high level of preparedness and screening response. Nurses, however performed at a statistically lower level on preparedness and screening. The proportion of clinicians (29.1%) who, on average, reported always or almost always conducting routine screening techniques was however, higher than a recent American study [14] which found only 15% of nurses working in college-based health services screened for DFV. Research on screening practices varies widely. For example, previous studies within paediatric and obstetric departments reported between 8.5 to 57% of doctors and nurses screen for DFV ([15]; Roush 2012). Variability may relate to setting, measures and data collection procedures. Furthermore, findings on this topic are difficult to compare as much of the literature reports percentage of women screened rather than screening practices of staff.

Higher levels of training were positively correlated with all screening and response practices. This was an expected finding and reflected previous studies that have shown DFV training and education increased the likelihood of clinicians screening for DFV [8, 16, 17]. Social work participants in the current survey reported the highest level of training than any other professional group and DFV screening and response practices. Participating social workers often worked in emergency, maternity and mental health departments (under the Division of Allied Health) and are usually engaged with patients following a referral from other clinicians who identified psychosocial issues or risk. As such, Social Workers were the experts who provided therapeutic interventions and support.

Nearly 35% of participants felt they did not have sufficient training to undertake DFV screening and response. Previous reviews have also found that healthcare providers lacked confidence and knowledge to address DFV when it was detected [18]. Lack of confidence has been successfully addressed in other settings through the introduction of screening protocols and clear guidelines covering a wide range of situations. While many of our participants (72%) also reported that guidelines and protocols were a significant enabler to screening, results from this research suggest that further support is needed to bolster clinician confidence. To better prepare health professionals experiential learning activities such as simulation; role-play, values-clarification and peer mentoring in the workplace may be beneficial. Screening and responding to DFV is more than ‘asking the question’. It requires empathy, trust, being attuned to non-verbal body language, and not blaming the person at risk or experiencing violence. Adopting a strengths-based approach can help women to disclose and take active steps to keep themselves and their children safe, even if they choose not to leave the relationship.

The major barriers to screening described in this study are commonly cited concerns. The presence of partners or relatives during consultations, women’s unwillingness to disclose, language barriers, time constraints, and lack of knowledge are common themes arising from prior research and have remained relatively unchanged for over 20 years [16, 19]. To some extent, these factors reflect the continued dominance of the medical model of care. Transitioning to a social model that places the woman at the centre of care, with a known carer (such as continuity of midwifery care models) may ameliorate some of these barriers to screening and response. This was illustrated in an Australian study with 210 women who reported they were more likely to discuss DFV with a known and trusted health care provider [4].

The current study highlights disparities across professional groups regarding DFV training, knowledge, and practice. At the participating site there was no expectation of DFV screening or response within key areas such as the Emergency and Mental Health Departments. A clear exception was Maternity services where screening for DFV is compulsory. A piecemeal approach across an organisation means that opportunities to detect and provide assistance to those experiencing DFV are missed. Findings confirm that nursing staff were less prepared or knowledgeable than midwives, social workers, and medical officers to undertake DFV screening and response. These findings suggest that policies, training, and procedures need to be implemented on a whole of organisation basis. At the participating site attending DFV training is not mandatory and likely to be undertaken by staff with an interest in DFV, or those who feel it would benefit their practice. An expectation of routine screening in all departments may increase the uptake of this education and improve staff practices and confidence.

While an in-depth response to DFV disclosure by a clinician may not be feasible in many practice contexts, new technologies within healthcare now allow for suspicions of DFV, or missed screening opportunities, to be followed up at later appointments or for an alert to be included in the electronic health record. Online patient notes and pro-forma questionnaires provide health care providers with easy access to screening tools. These tools are of benefit to clinicians and patients and should be utilised along with education. While education is beneficial to DFV detection, previous studies have found that education alone is not as effective as approaches that incorporate other resources, such as screening protocols and standardised questionnaires [20].

There are some limitations to the study that should be taken into consideration. The small numbers within each of the different professional groups may have introduced error when analysing DFV screening and response items. Scores for perceived preparedness, knowledge, and practices may have been affected by the varying degree of patient engagement and level of prior DFV involvement of participants. For this reason, a large portion of the sample did not answer questions relating to screening and response practices. Further research into the knowledge, competencies and training needs of staff in different clinical divisions may be beneficial to develop successful training and education for these groups. Further, questions as to the nature of their patient engagement and level of DFV screening and response expected in their role would be beneficial to gain insight into the needs of the population and points of engagement for patients experiencing DFV that could be targeted for improved patient care and staff training. All scales were new and although internal reliability was good, exploratory factor analysis of the scales would be useful in refining the number of items due to low factor loadings and redundancy. The survey was voluntary and self-report. It could be that participants were interested in DFV and their practices differed from staff who did not respond. Future research could consider other indicators of quality practice – such as observation, audits of medical records, and training by simulation.

## Conclusions

Clinicians who had received high levels of training were more likely to feel prepared and enabled to undertake DFV screening and response practices. Our findings reinforce the importance of regular ongoing training and education for staff. Major barriers were the presence of partner and language barriers. While written protocols and a supportive work environment were the principal enablers to screening, organisation-wide protocols are required for consistency across departments and professions. Even though political and public desire for change is high, and many programs are being implemented nationally and internationally to address DFV, the same problems that have existed for decades remain. Improved DFV education for all clinicians is still requested by staff, DFV knowledge and confidence remains lower than desirable, and consistent screening tools, guidelines and protocols for screening and referral are frequently lacking in health care services. While improvements have been made, more needs to be done to improve services for the detection, care, and prevention of abuse for those experiencing DFV.

## Data Availability

The datasets used and/or analysed during the current study available from the corresponding author on reasonable request.
