# Correction to: Australian hospital staff perceptions of barriers and enablers of domestic and family violence screening and response

**DOI:** 10.1186/s12913-021-07207-4

**Published:** 2021-11-08

**Authors:** Debra K. Creedy, Kathleen Baird, Kerri Gillespie, Grace Branjerdporn

**Affiliations:** 1 Transforming Maternity Care Collaborative, School of Nursing and Midwifery, Griffith University, University Drive, Meadowbrook, Queensland 4131 Australia; 2 Gold Coast University Hospital, Parklands Drive, Meadowbrook, 4215 Australia; 3 Centre for Midwifery, Child and Family Health, School of Nursing and Midwifery, Faculty of Health, University of Technology Sydney, Ultimo, 2007 Australia

Correction to: *BMC Health Serv Res* **21,** 1121 (2021). https://doi.org/10.1186/s12913-021-07083-y

Following publication of the original article [1], the authors identified an error in the family name of the author Grace Branjerdporn.

The incorrect author names are: Grace Brandjerdporn.

The correct author names are: Grace Branjerdporn.

The author group has been updated above and the original article [1] has been corrected.
